# An Innovative Huffman Forest-Based Method to Detected Railroad Station Anomalies

**DOI:** 10.3390/s22103915

**Published:** 2022-05-22

**Authors:** Yuan Wang, Xiaopeng Li

**Affiliations:** Department of Civil and Environmental Engineering, University of South Florida, Tampa, FL 33612, USA; yuanwang2@usf.edu

**Keywords:** anomaly detection, Huffman forest, railroad station

## Abstract

Detecting railroad station anomalies is a critical task prior to segmentation and making optimization decisions for each cluster. Three types of anomalies (local clustered, axis paralleled, and surrounded by normal instances) caused by the specialty of railroad operations bring the existing methods non-trivial challenges in detecting them accurately and efficiently. To tackle this limitation of existing methods, this paper proposes a novel anomaly detection method named Huffman Anomaly Detection Forest (HuffForest) to detect station anomalies, which leverages Huffman encoding to measure abnormalities in certain railroad scenarios with high accuracy. The proposed method establishes a Huffman forest by constructing trees from the perspective of data points and subsequently computes anomaly scores of instances considering both local and global information. A sampling-based version is also developed to improve scalability for large datasets. Taking advantage of the encoding mechanism, the proposed method can effectively recognize the underlying patterns of railroad stations and detect outliers in various complicated scenarios where the conventional methods are not reliable. Experiment results on both synthesized and public benchmarks are demonstrated to show the advances of the proposed method compared to the state-of-the-art isolation forest (iForest) and local outlier factor (LOF) methods on detection accuracy with an acceptable computational complexity.

## 1. Introduction

The continued and substantial growth in railroad transportation in many countries indicates that railroad operations optimization remains a vital issue for decision-makers [1]. While seeking to maintain acceptable levels of service, railroad operators must make the best possible use of available infrastructures and capacities. Significant returns of investment are always achieved by optimizing the operations of network nodes—junctions and stations—that usually form the capacity bottlenecks on the system [2].

Subject to resource constraints, such as expertise, investments, and management capabilities, it is usually regarded as impractical engineering to make a dedicated optimization strategy for each station. A more realistic and cost-effective way is to segment stations into several classes according to their performances and make diversified strategies for each class. For instance, the Chinese railroad has thousands of stations with different features such as trains throughout, origination train count, destinations train count, passenger population within 50 m, passenger volume, platform count, terminal house squares, connecting direction count, LBS information, etc. These stations are usually divided into three levels (e.g., premier-class, first-class, and second-class) according to traffic or four levels (e.g., super large, large, medium, and small) according to the number of platforms [3,4]. Then, decision-makers will make different management requirements, objectives, and optimization strategies for each class.

Obviously, the quality of segmentation will directly contribute to optimization performance. Regardless of segmentation techniques, prior to segmentation, one critical but challenging part is to detect outliers, which can happen in the railroad operations due to the complex infrastructure, such as the track [5] and overhead system [6]. These outliers are usually special cases that can hardly be covered by any general strategy and should be analyzed case-by-case and treated delicately. For example, Wuchang rail station is located at the intersection of four high-volume rail lines and has an extremely high traffic volume but a very limited number of platforms. On the one hand, if it was clustered into the high-volume class, the optimization strategy may not be effective due to the limited infrastructure. On the other hand, if it was included in the few-platforms class, the low priority setting of this class would bring many significant operation troubles due to the high-volume fact. Furthermore, this station is an isolated case caused by its location and historical reality, thus it is managerially unnecessary to create a particular high-volume-but-few-platform class. Hence, it should be identified as an anomaly to receive a dedicated treatment.

Thanks to pioneering studies, many great measurement tools have been developed to provide insights into railroad performance for decision-makers [7,8,9]. These measurement frameworks provide various attributes of station performance. In addition, many studies proposed various classification methods from different perspectives, ranging from traffic volume, passenger frequency to facility condition [10,11,12,13,14]. However, to our best knowledge, no studies focus on detecting station anomalies considering both traffic and infrastructure features.

Anomaly detection is a long-existing research topic, which focuses on identifying the data points (usually referred to as instances) whose distributions differ from the majority. Since anomaly usually implies important information, this technique is widely utilized in a variety of fields to discover outliers, thereby recognizing the abnormal phenomenon, which significantly helps the researchers in multiple real-world applications such as avoiding potential risks and capturing new observations [15].

Accurately and efficiently detecting the anomaly is usually a non-trivial task and a large number of studies have been devoted to this line of research. The existing approaches for anomaly detection can be classified into supervised approaches and unsupervised approaches, where unsupervised approaches can be further divided into distance-based methods, density-based methods, and model-based methods. Since large railroad operators usually manage thousands of rail junctions or stations [16], which makes the labels of anomalies difficult to be obtained, we focus on unsupervised approaches in this work.

Most of the existing distance-based anomaly detection techniques identify the abnormal instances based on the assumption that an outlier lies far away from the major data distribution, and is consequently subject to a greater distance to most of the others [17]. However, this series of techniques are proven to be effective only for certain types of outliers since they only focus on the global view of the dataset [18,19]. Therefore, their detection capability is limited for more complex structures that widely exist in the real world.

In order to overcome these drawbacks of distance-based techniques, density-based measurements have been proposed to improve the capability for recognizing the "local” outliers that lie in the low-density area [18]. Although this type of method is proven to be more effective on local outliers, there are still unsolved issues. For instance, low density does not always imply outliers especially when it is measured considering only the local context [20]. Furthermore, both density-based and distance-based methods may easily fail if there are regions with different densities in a dataset [20].

Apart from distance-based and density-based methods, the machine learning algorithms originally developed for other tasks have also been leveraged for anomaly detection as model-based detection methods, such as support vector machine (SVM) and random forest [21,22]. However, their abilities on anomaly detection are in fact a byproduct of the mechanisms designed to fit other scenarios and have not been optimized to detect anomalies [20]. This induces the limitation that the efficacy of a model-based method on anomaly detection critically depends on the similarity between the current and original tasks.

In contrast to the aforementioned model-based techniques, the isolation tree (iForest) is originally designed for anomaly detection. It constructs isolation trees that split the instances based on attributes values [20,23]. Compared to normal instances, the outliers are more likely to be separated in the branches close to the roots. The isolation trees are subsequently assembled to form an iForest, which measures the level of abnormalities of each instance based on the average length of paths. Compared to the existing distance-based and density-based methods such as ORCA [24] and LOF [18] as well as other model-based methods such as one-class SVM [21] and random forest [22], iForest has demonstrated its advantages for both detection accuracy and efficiency.

**Figure 1:**
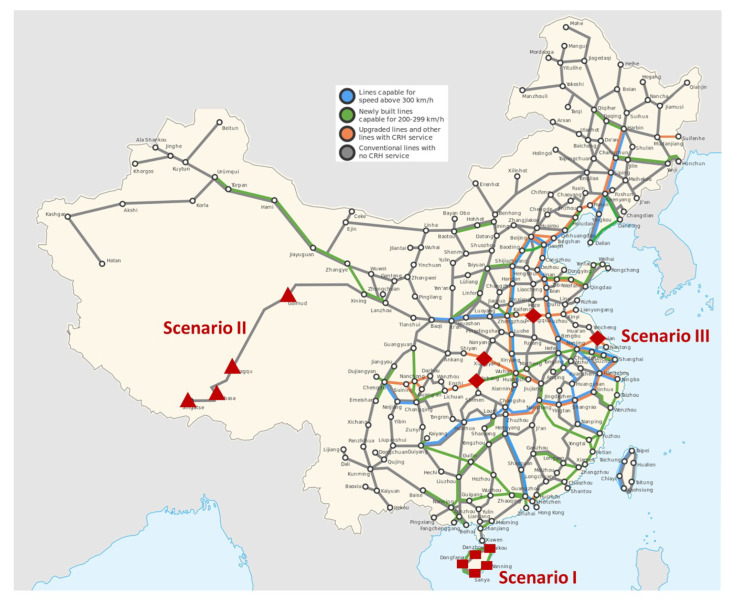
Railroad (passenger) network of China—2016 version.

Although iForest is recognized as a state-of-the-art method, there are still several types of anomalies caused by the railroad station operations that cannot be effectively detected by it. Since stations are nodes in the railroad network that are connected by lines, some of their attributes are not independent of each other and highly impacted by the network structure. Figure 1 presents the simplified passenger railroad network of China, where colored lines represent the rail lines and dots represent the stations. Three commonly seen scenarios are as follows:

1. **Local clustered anomalies**, which are the local anomalies that form a cluster. Square marks in Figure 1 represent five stations on Hainan island, where they compose a local network aiming at satisfying local demands and have a very weak connection to the mainland network due to the channel. They are clustered locally because their distributions of traffic and demand attributes are similar to each other and identified as anomalies since these distributions significantly differ from the majority.
2. **Axis paralleled anomalies**, which are the anomalies with identical values in some attributes, thus parallel to the axis. Triangle marks in Figure 1 represent four stations in the Tibet part of the Qinghai-Tibet line, which is located at the edge of the rail network. Four stations are parallel to the traffic axis since they are in the same line without any other branches and share the exact same traffic distributions, regardless of their possible variation in demands. They are further considered anomalies since their traffic is much lower than the majority.
3. **Anomalies surrounded by normal instances**. Since most of the China high-speed rail lines were newly built rather than upgraded from old lines, the function of stations is very distinguishable as they focus on either high or regular speed trains, and the distribution of average speed passing through stations follows a scattered pattern—either low or high. However, four stations with the diamond mark in Figure 1 are conjunctions of both high and regular speed lines and their functionalities are hybrid for both types of trains so that their average speed distributions concentrate in the middle between low and high. So, they can be regarded as anomalies (with concentrated features) surrounded by normal instances (with scattered features)

Due to the importance of these anomalies in real-world scenarios, efforts have been devoted to discovering the corresponding solutions. The work [15] has involved a split-selection mechanism in the original version of iForest and transformed it into SCiForest for the detection of local clustered anomalies. The work [25] leverages the advantages of both iForest and distance-based methods by adopting nearest neighbor distance in the space partitioning framework, which can successfully detect both local clustered anomalies and the anomalies surrounded by normal instances with high efficiency. Despite the advances of recent research, no existing method can tackle the aforementioned three issues simultaneously.

In order to overcome these difficulties, we have reconsidered the characteristics of outliers and we sitll find distance an effective measurement of abnormality level if utilized in an appropriate way. Thus, the proposed method first computes the distances between each pair of instances and subsequently evaluates the abnormality of each instance using both global and local information extracted from the distances. For an instance, the global information refers to its abnormality level observed by other instances based on the distances and the local information refers to the abnormality levels of other instances from the perspective of itself, which can reflect the abnormality level of its own in return. In order to effectively transfer the distance information into abnormality levels, Huffman encoding is employed to accurately identify the remote distances, which usually lie in the rare region of all distances. Thus, a Huffman tree is built for each data instance and thereby forms a Huffman forest for the dataset. Subsequently, an anomaly score is computed based on the encoding depths of the Huffman trees. Considering the nature of the proposed method, it is named **Huffman Anomaly Detection Forest (HuffForest)**.

It should be noted that detection accuracy is usually given a much higher priority than computation complexity since rail stations are critical assets for railroad companies and optimization investments tend to be massive and long-term. Therefore, although the proposed method is limited by the high time complexity since the distances between all pairs of instances need to be computed, the strengths of detecting anomalies with higher accuracy can still be taken advantage of. In addition, in order to minimize the weakness of time efficiency, a sampling-based version of HuffForest was also developed. It computes the anomaly score of an instance using a subset of instances created by an innovative stratified sampling mechanism. This significantly improves the scalability for processing large datasets without sacrificing detection accuracy.

In order to validate the efficacy of HuffForest, four synthetic benchmarks were created to represent the above facts of railroad networks. These benchmarks contain local clustered anomalies, axis paralleled anomalies, and anomalies surrounded by normal instances, respectively. While these scenarios cannot be simultaneously handled by existing anomaly detection techniques, HuffForest can successfully detect the anomalies for all these benchmarks. Furthermore, in order to better demonstrate the strength of the proposed methods, the original and sampling-based HuffForest are also compared with LOF and iForest on 15 public benchmarks, which shows a superior detection accuracy in 14 of them with acceptable computational costs. Thus, the contributions of this work are summarized as follows.

- An innovative Huffman forest anomaly detection method (HuffForest) is proposed in this work, which can tackle the limitations of the existing anomaly detection techniques when they are utilized in similar scenarios of rail station anomaly detection.
- A sampling-based version of the HuffForest method is developed to improve time efficiency and provide a scalable solution for large datasets.
- Experiments are conducted to validate the proposed Huffman forest-based anomaly detection methods, where the proposed methods outperform existing methods on detection accuracy in 14 public benchmarks with acceptable computational cost.

The remainder of this paper is organized as follows. The proposed Huffman forest anomaly detection method is presented in Section 2 and a sampling-based version of HuffForest is developed in Section 2.2. Experiments are conducted to evaluate the proposed methods in Section 3. Finally, we conclude in Section 4.

## 2. Proposed Method

In this section, a HuffForest method and its sampling version are proposed and presented with details. It should be noted that, although the proposed method is inspired by the specialty of railroad station operations, it is a general anomaly detection algorithm that can be applied in any area but is expected to show superior capabilities to handle three types of anomalies—local clustered anomalies, axis paralleled anomalies. To present its performance in diverse areas, numerical analysis on public benchmarks is conducted in Section 3.

### 2.1. Huffforrest

Consider a set of instances denoted by ${\left\{x_{i}\right\}}_{1\leq i\leq n}$ while $x_{i}\in R^{m}$, where *m* is the number of dimensions of each instance. The target of this work is to identify the abnormal instances based on the topological information. Hence, the distances between all pairs of instances are computed while the distance between $x_{i}$ and $x_{j}$ is denoted by $d_{ij}$. Other than directly computing the distances, we first normalize the instances to mitigate the impact induced by the differences of distributions between each dimension. The normalized version of an instance $x_{i}$ is represented by ${\bar{x}}_{i}$, in which the *k*-th dimension is computed by

(1) $${\bar{x}}_{ik}=\frac{x_{ik}-\mu_{k}}{\sigma_{k}},\;\forall 1\leq i\leq n,\;1\leq k\leq m$$

where $\mu_{k}$ and $\sigma_{k}$ are the mean and standard deviation of dimension *k* over the dataset, respectively. Subsequently, the distance between instances *i* and *j* is computed as the Euclidean distance between ${\bar{x}}_{i}$ and ${\bar{x}}_{j}$,

(2) $$d_{ij}={∥{\bar{x}}_{i}-{\bar{x}}_{j}∥}_{2},\;\forall 1\leq i,\;j\leq n$$

The most critical part of the proposed method is the way to effectively utilize the distances. Based on the assumption that the abnormal instances are those remote from the major distribution, a natural idea is to measure the “remote level” of each instance and identify the abnormal ones accordingly. Unlike the existing distance-based approaches, this work identifies the abnormal instances using a voting-based method since we have the following two observations.

- For a normal instance, the range of distances from it to others is relatively wide since normal instances can be close to it, whereas abnormal instances can be far away from it.
- For an abnormal instance, the range of distances from it to others tends to be narrow since most instances are normal ones which are usually remote from it.

Based on the aforementioned observations, each instance can create its own view of the others in the dataset and the abnormality level of an instance can be measured using both global information and local information. For an instance, global information refers to the evaluation of it created by the others and local information refers to its evaluation of the others in the dataset. Global and local information can be jointly considered to form the abnormality measurement for each instance.

At this step, a crucial task is to find an appropriate representation of the measurements. In order to reach this target, Huffman encoding [26] is employed in this work since it can use the encoding length to represent the frequency of appearance. Given a set of symbols, Huffman encoding represents them using variable-length codes based on frequency-of-occurrence for efficient information compression such that a symbol with higher frequency is represented by a code with a shorter length [26]. In Huffman encoding, each symbol to be encoded is treated as a node and the two nodes with the minimum frequencies are iteratively grouped as a new node, which forms a binary tree structure, referred to as “Huffman Tree”. Following this logic, a longer distance can be projected to a longer encoding length since it is usually a rare event. Thus, in order to measure the abnormality level of an instance, a natural idea is to form a Huffman tree from the perspective of a specific instance (termed as reference instance), in which all the instances in the dataset except reference instance are treated as symbols. For a dataset, Huffman trees are generated from the perspective of each instance, which forms a Huffman forest that combines the evaluation of the dataset illustrated by each tree.

Considering the original definition of Huffman tree, we use frequencies-of-occurrence derived from distances as the weights of instances. To be specific, while constructing a Huffman tree based on a reference instance, the instances whose distances to the reference instance lie in a high-density range are those with high frequencies-of-occurrences. In order to implement this idea, the distances need to be discretized to appropriately compute frequencies-of-occurrence before being encoded using Huffman forest. In order to guarantee that the distances can be discretized using a unified criterion, we further normalize the distances for each potential Huffman tree as in Equation (3) such that it is distributed between 0 and 1.

(3) $$d_{i}^{*}\left(x_{j}\right)=\frac{d(x_{i},x_{j})}{\sum_{j=1}^{n}d\left(x_{i},x_{j}\right)},\;\forall 1\leq i,j\leq n$$

where *i* refers to a reference instance. Subsequently, $d_{i}^{*}\left(x_{j}\right)$ are discretized using a stride of 0.001. Subsequently, we can compute the frequencies-of-occurrences by directly counting the frequencies of discretized distances and establish a Huffman tree for each instance.

After the Huffman trees are established, we denote the encoding length of instance j∃j≠i by $L_{i,j}$ in Huffman tree *i*. Finally, the anomaly score of an instance *i* is computed by

(4) $$score_{i}=\log\left(\frac{\sum_{j=1}^{n}L_{i,j}}{\sum_{j=1}^{n}L_{j,i}}\right),\;\forall 1\leq i\leq n$$

In Equation (4), the nominator represents the global information that the abnormality level is high if an instance is remote from other instances and the denominator represents the local information that the abnormality level is high if its distances from other instances are all located in a high-frequency range and resulting in relatively short encoding lengths. Given the dataset, the end-to-end procedure for computing the anomaly scores of all instances is depicted in Algorithm 1. Note that the distances between all instance pairs need to be computed in order to build the Huffman forest, and hence, the time complexity of the proposed method is $O(n^{2})$. This will limit the application of the proposed method on very large datasets. To tackle this issue, a sampling-based version of HuffForest is also presented in this work.
**Algorithm 1:** HuffForest Anomaly Detection**Input:**${\left\{x_{i}\right\}}_{1\leq i\leq n}$ **Output:**$score_{i}\;\forall 1\leq i\leq n$Normalize all dimensions for each instance $x_{i}\in {\left\{x_{i}\right\}}_{1\leq i\leq n}$according to Equation (1).Compute the distance between each pair of instances *i* and *j* as $d_{ij}$according to Equation (2).For each instance *i*, compute the normalized distances $d_{i}^{*}\left(x_{j}\right)$according to Equation (3).For each instance *i*, build the Huffman tree for the rest instances with frequencies-of-occurrence created by counting $d_{i}^{*}\left(x_{j}\right),\forall j\neq i$.Denote the encoding length of instance j∃j≠i by $L_{i,j}$ in Huffman tree *i*.Compute the abnormality level of each instance *i* as $\;score_{i}$according to Equation (4).return$\;score_{i}\;\forall 1\leq i\leq n$

### 2.2. Sampling-Based Huffforest

In order to overcome the difficulties induced by the high computational complexity of HuffForest, a sampling-based version was further developed to identify anomalies based on a subset of the original dataset. While sub-sampling can substantially reduce the time complexity, it does not affect the capability for anomaly detection if the sampling does not change the data distribution. Furthermore, it is also proven to be an effective approach to tackle masking and swamping effects [20,23,27]. While masking and swamping are common problems of anomaly detection induced by the increasing of normal and abnormal instances [23], sub-sampling can reduce the number of instances considered for anomaly detection, thereby mitigating the masking and swamping effects.

In order to design an effective sampling mechanism, a critical issue is to preserve the distribution of the original dataset. Although a naive strategy can be uniformly sampling based on the spatial distribution of the instances, it is sensitive to the variation of density. Thus, we develop a novel distribution-preserving sampling strategy to accelerate the proposed HuffForest method while maintaining the detection accuracy. Given a dataset, it first computes the geometric center of all instances, denoted by

(5) $$\vec{O}=\left(\frac{\sum_{i}^{n}x_{i1}}{n},\frac{\sum_{i}^{n}x_{i2}}{n},\ldots ,\frac{\sum_{i}^{n}x_{im}}{n}\right),$$

where $x_{ik}$ is the *k*-th attribution of instance $x_{i}$. Subsequently, the distance from each instance *i* to $\vec{O}$ is computed as

(6) $$d_{o}\left(i\right)={∥x_{i}-\vec{O}∥}_{2},\;\forall 1\leq i\leq n$$

The instances are then sorted according to $d_{o}\left(i\right)$ and uniformly arranged into *B* bins. Subsequently, ψ instances are sampled uniformly from the bins to construct a subset for identifying abnormal instances. In order to detect all the potential abnormal instances, the dataset is repeatedly sampled without putting back until no instances are left. Since a subset needs a sufficient number of instances to represent the distribution, if the number of instances in the dataset is less than 2·ψ after certain sampling steps, all of them are selected in the last step to avoid the bias induced by insufficient sub-sampling. Given the dataset, the end-to-end steps of sampling-based HuffForest are depicted by Algorithm 2. Since the Huffman forest-based anomaly detection is applied on each subset, the time complexity is reduced to $O\left(\frac{n}{\psi }\psi^{2}\right)=O\left(n\psi \right)$.
**Algorithm 2:** Sampling-Based HuffForest
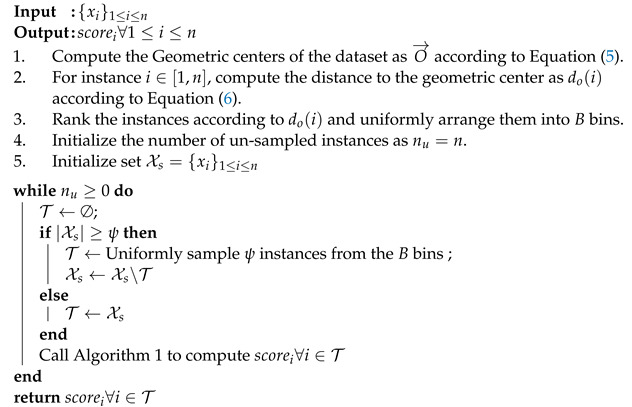


## 3. Experimental Results

In this section, the proposed HuffForest method is evaluated on detection accuracy and time efficiency. Two existing techniques, iForest and LOF are used as baselines to demonstrate the advantages of the proposed methods. The experiments are conducted on a 2.10 GHz Linux cluster with 512 GB RAM. We implement HuffForest using Python 3.6 and directly call “scikit-learn” package for iForest and LOF. The detection accuracy and time efficiency are evaluated by AUC and time of computation, respectively.

The HuffForest method is evaluated and compared to baseline methods using two sets of experiments. In the first set of experiments, the aforementioned methods are analyzed in details on four synthetic datasets to demonstrate the capability of the proposed method on tackling the difficult cases. In the second set of experiments, the aforementioned methods are compared on a variety of public benchmarks from OpenML.com to demonstrate the advantage of the proposed method on detection accuracy.

### 3.1. Performance on Synthetic Data

This section is to evaluate the capability of the proposed method on three specific types of anomalies. Without losing generality, the proposed method and the-state-of-art techniques are tested on four two-dimension synthetic datasets. It should be highlighted that synthetic datasets were used to create four scenarios separately so that all methods can be evaluated respectively.

With prior knowledge of railroad structure, four synthetic datasets were generated to represent three scenarios mentioned in Section 1 that contain the types of anomalies which are difficult for existing techniques.

**Figure 2:**
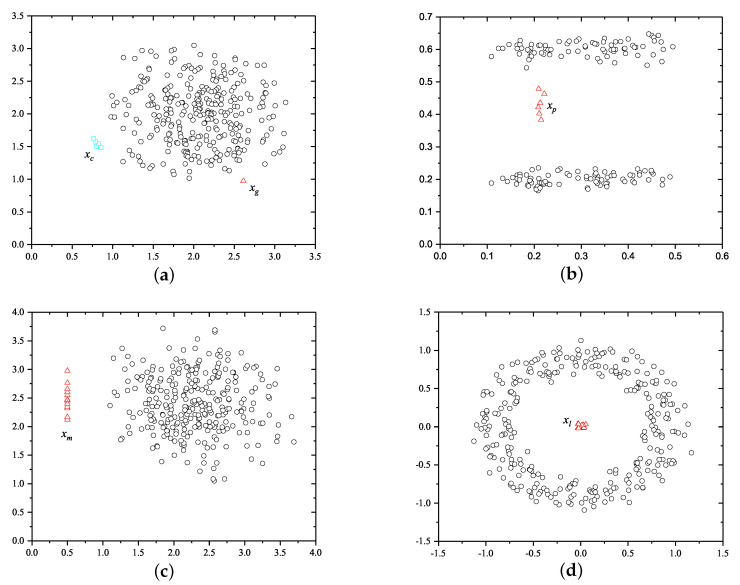
Four synthetic datasets that represent local clustered anomalies, anomalies parallel to the axis, and anomalies surrounded by normal instances. (**a**) Dataset with local clustered anomalies and local scattered anomalies. (**b**) Dataset with local anomalies parallel to the axis. (**c**) Dataset with global anomalies parallel to the axis. (**d**) Dataset with anomalies surrounded by normal instances.

- The dataset corresponding to Figure 2a is generated according to the local clustered anomaly scenario like Scenario I, where the majority of samples are generated subject to one distribution whereas some clustered anomalies are generated subject to a significantly different distribution.
- The datasets corresponding to Figure 2b,c are synthesized to evaluate the capabilities of detecting anomalies parallel to the axis which is the scenario similar to Scenario II, where anomalies are generated to be parallel to the horizontal axis and the normal instances are generated away from anomalies.
- The dataset corresponding to Figure 2d is synthesized to demonstrate the case of anomalies surrounded by normal instances referring to the scenario in Scenario III, where the anomalies are clustered in the center surrounded by the majority samples with remarkable distances.

The detection results are shown in Figure 2, and Table 1, Table 2, Table 3 and Table 4, respectively. In terms of LOF, two different search ranges (k=10 and k=15) are considered for a fair comparison.

Figure 2a and Table 1 show a dataset with seven local clustered and one local scattered anomaly. As shown in the experimental results, the anomalies are ranked at the top eight by Huffman forest. On the contrary, the baseline methods can only detect the scattered local anomaly and none of them can correctly detect the clustered local anomalies.

In order to demonstrate the capability of the Huffman forest on the detection of anomalies parallel to the axis, two datasets are generated as shown in Figure 2b,c. Figure 2b depicts a dataset consisting of 82 instances with six anomalies located between two normal instance clusters. As shown in the experimental results in Table 2, Huffman forest and LOF with both configurations can rank the anomalies at the top of the anomaly list while the abnormal instances are ranked much lower in the list of iForest.

Figure 2c depicts a dataset of 313 instances with 13 anomalies remote from the main cluster of normal instances. As shown in the results in Table 3, the abnormal instances are ranked at the top in the list of Huffman forest while they are ranked much lower in the list of other approaches.

In Figure 2d, a dataset consisting of 311 instances including 11 anomalies is surrounded by the normal instances. As shown in the experimental results in Table 4, both Huffman forest and LoF with k=15 can rank the abnormal instances at the top of anomaly lists while other methods cannot generate an accurate detection result. Overall, Huffman forest demonstrates a superior detection capability on all the considered difficult scenarios while none of the baseline methods can tackle them simultaneously.

### 3.2. Performance on Public Benchmarks

Although the proposed method outperforms on datasets synthesized according to railroad knowledge, evidence of advances will be strengthened if it shows superiority on a wider range of anomaly detection. Thus, the HuffForest method is further evaluated on the public benchmarks from OpenML.org that are widely adopted in the domain of anomaly detection in this part. The original version of HuffForest is compared to iForest and LOF on 12 benchmarks as shown in Table 5. In this set of experiments, HuffForest has no hyper-parameter to tune. For iForest, we keep the default configuration such that the iForest is consists of iTrees while each iTree is built using 256 attributes. In order to guarantee the fairness of comparison, we run iForest 30 times on each dataset, and the trial with the largest AUC is picked. For LOF, we fix the search range as k=10. We can observe from the table that only on Musk dataset Huffman forest (AUC =0.99) is slightly inferior to iForest (AUC =1). On the other 11 benchmarks, it leads to detection accuracy. Although the time for computation is longer than the baseline methods, it is still within an acceptable range.

The sampling-based version of HuffForest is evaluated on three large benchmarks with over 200,000 instances. We select the sub-sampling size as ψ=150 and the number of bins as B=10 for the sampling-based HuffForest and keep the configurations of baseline methods the same as the last set of experiments. The results are shown in Table 6. We can observe from the table that sampling-based HuffForest outperforms the baseline methods on all these benchmarks. Although it needs a longer computational time, the sampling-based Huffman forest offers a reasonable choice for the applications where detection accuracy is more critical, which is usually the case of railroad station operations optimization.

## 4. Conclusions

In this paper, we proposed a Huffman forest-based anomaly detection (HuffForest) method that tends to overcome three typical scenarios of identifying station outliers according to their performance. The proposed method leverages Huffman encoding to measure the abnormality of the dataset from the perspective of each instance. The measurements are subsequently divided into global measurements and local measurements, which are jointly considered to compute the anomaly score of each instance. Furthermore, a sampling-based version of HuffForest is developed to reduce the time complexity without sacrificing detection accuracy. Experiments are conducted to validate the proposed HuffForest method. The experimental results on four synthetic datasets representing the railroad scenarios proved that HuffForest can generate robust detection results in the scenarios where existing methods are not reliable. The proposed method is also compared to the state-of-the-art iForest and LOF methods on multiple classic benchmarks, where it is demonstrated to outperform the baseline methods on detection accuracy with an acceptable computational complexity.

## Figures and Tables

**Table 1:** A total of 308 instances are generated in this example including 8 anomalies (7 local clustered anomalies and 1 local scattered anomaly). Among the three detection methods, the AUC of HuffForest is 1, which means that it ranked all anomalies at the top.

Ranking	$x_{c}$	$x_{g}$
huffForest	1–7	8
iForest	10, 105, 12, 27, 29–32	1
LOF (*k* = 10)	41, 47, 52, 56, 65, 67, 82	1
LOF (*k* = 15)	42, 45–47, 54, 57, 59	1

**Table 2:** In this example, six anomalies are generated between the normal clusters in parallel with the y-axis. HuffForest and LOF (with both k=10 and k=15) can rank the anomalies at the top of the list. However, iForest cannot make an accurate detection.

Ranking	$x_{p}$
huffForest	1–6
iForest	7, 11, 13, 18, 19, 25
LOF (*k* = 10)	1–6
LOF (*k* = 15)	1–6

**Table 3:** In this example, 13 anomalies are generated in parallel with the y-axis. HuffForest is the only method that ranks the anomalies at the top of the list.

Ranking	$x_{m}$
huffForest	1–13
iForest	2, 10, 13, 14, 18, 20, 22, 26, 27, 31, 35, 37, 39
LOF (*k* = 10)	20, 92, 113, 176, 181, 206, 227, 234, 272, 283, 286, 287, 310
LOF (*k* = 15)	49, 57, 72–82

**Table 4:** In this example, the normal instances form a ring, and 11 anomalies are surrounded by this ring. As shown in the detection results, HuffForest and LOF with k=15 can rank the anomalies at the top while other methods rank them much lower.

Ranking	$x_{l}$
huffForest	1–11
iForest	125, 147, 148, 151, 159, 164, 170, 187, 197, 199, 207
LOF (*k* = 10)	86, 135, 141, 161, 171, 173, 183, 184, 233, 267, 268
LOF (*k* = 15)	1–11

**Table 5:** HuffForest (HF), iForest (iF) and LOF are compared on 12 benchmark datasets. The best detection accuracies (measured by AUC) are emphasized with bold symbols.

	N	d	Anomalies	AUC			Time (Second)		
				HF	iF	LOF	HF	iF	LOF
Mnist	7603	100	700 (9.2%)	1.00	0.83	0.58	217.00	0.95	8.56
Satimage-2	5803	36	71 (1.2%)	1.00	1.00	0.59	122.37	0.53	1.07
Optdigits	5216	64	150 (3%)	1.00	0.79	0.62	112.50	0.60	2.96
Musk	3062	166	97 (3.2%)	0.99	1.00	0.39	47.43	0.56	1.41
Cardio	1831	21	176 (9.6%)	0.98	0.94	0.60	22.50	0.25	0.10
Letter	1600	32	100 (6.25%)	1.00	0.65	0.91	17.57	0.25	0.11
Vowels	1456	12	50 (3.4%)	1.00	0.80	0.95	15.63	0.24	0.03
Ecoli	336	7	9 (2.6%)	0.90	0.87	0.85	2.39	0.16	0.00
WBC	278	30	21 (5.6%)	1.00	0.96	0.89	2.84	0.17	0.01
Vertebral	240	6	30 (12.5%)	0.94	0.41	0.49	1.73	0.14	0.00
Glass	214	9	9 (4.2%)	0.84	0.74	0.78	1.60	0.14	0.00
Wine	129	13	10 (7.7%)	0.95	0.88	0.94	0.79	0.14	0.00

**Table 6:** Sampling-based HuffForest (S-HF), iForest (iF) and LOF are compared on three large benchmark datasets. The best detection accuracies (measured by AUC) are emphasized with bold symbols. S-HuffForest refers to sampling-based HuffForest.

	N	d	Anomalies	AUC			Time (Second)		
				S-HF	iF	LOF	S-HF	iF	LOF
Http	567,479	3	2211(0.4%)	1.00	1.00	0.36	6920.40	28.33	93.61
F-Cover	286,048	10	2747(0.9%)	0.91	0.88	0.57	2782.09	13.55	4.68
Mulcross	262,144	4	26,214(10%)	1.00	0.97	0.58	2512.26	14.17	3.81

## Data Availability

Not applicable.
